# Requirement of the CXXC Motif of Novel *Francisella* Infectivity Potentiator Protein B FipB, and FipA in Virulence of *F. tularensis* subsp. *tularensis*

**DOI:** 10.1371/journal.pone.0024611

**Published:** 2011-09-08

**Authors:** Aiping Qin, David W. Scott, Meaghan M. Rabideau, Emily A. Moore, Barbara J. Mann

**Affiliations:** 1 Department of Medicine, University of Virginia, Charlottesville, Virginia, United States of America; 2 Department of Microbiology, University of Virginia, Charlottesville, Virginia, United States of America; 3 Office of Laboratory Management, Chinese Center for Disease Control and Prevention, Beijing, Peoples Republic of China; Université de Genève, Switzerland

## Abstract

The lipoprotein encoded by the *Francisella tularensis subsp. tularensis* locus FTT1103 is essential for virulence; an FTT1103 deletion mutant is defective in uptake and intracellular survival, and mice survive high dose challenges of greater than 10^8^ bacteria. This protein has two conserved domains; one is found in a class of virulence proteins called macrophage infectivity potentiator (Mip) proteins, and the other in oxidoreductase Disulfide Bond formation protein A (DsbA)-related proteins. We have designated the protein encoded by FTT1103 as FipB for *Francisella* infectivity potentiator protein B. The locus FTT1102 (*fipA*), which is upstream of *fipB*, also has similarity to same conserved Mip domain. Deletion and site-specific mutants of *fipA* and *fipB* were constructed in the Schu S4 strain, and characterized with respect to intracellular replication and *in vivo* virulence. A nonpolar *fipA* mutant demonstrated reduced survival in host cells, but was only slightly attenuated *in vivo*. Although FipB protein was present in a *fipA* mutant, the abundance of the three isoforms of FipB was altered, suggesting that FipA has a role in post-translational modification of FipB. Similar to many DsbA homologues, FipB contains a cysteine-any amino acid-any amino acid-cysteine (CXXC) motif. This motif was found to be important for FipB's role in virulence; a deletion mutant complemented with a gene encoding a FipB protein in which the first cysteine was changed to an alanine residue (AXXC) failed to restore intracellular survival or *in vivo* virulence. Complementation with a gene that encoded a CXXA containing FipB protein was significantly defective in intracellular growth; however, only slightly attenuated *in vivo*.

## Introduction

*Francisella tularensis* subspecies *tularensis*, also known as type A *Francisella*, causes a potentially life-threatening disease called tularemia. Tularemia can be contracted by the bite of an arthropod vector, or through inhalation of contaminated particles or bacteria. Concerns over use of *F. tularensis* as a biological weapon have arisen due to its documented use as a bioweapon in WWII, and reports of the development of weaponized strains that are resistant to antibiotics and vaccines [1], [2]. These concerns have led to increased interest in defining the mechanisms of virulence, and immunity as means towards identifying new targets for therapy and immune protection. There are several other less virulent subspecies and species of *Francisella* including the Live Vaccine Strain (LVS), an attenuated strain of *F. tularensis subsp. holarctica* that has been used as a protective vaccine in some parts of the world, but has never been licensed in the United States [3].

*F. tularensis* is a facultative intracellular bacterium that can invade a variety of cell types including macrophages, endothelial cells, and hepatocytes [4], [5], [6]. After phagocytosis *F. tularensis* resides in a phagosome for up to 4 hours before escaping to the cytoplasm [7], [8], [9]. Once in the cytoplasm bacteria replicate, induce apoptosis or pyroptosis, and are eventually released from cells. *F. tularensis* can also reenter the endocytic pathway and reside in a large membrane-bound compartment that has characteristics of an autophagocytic vacuole [10]. Thus far only a few loci have been directly implicated in phagosome survival or escape. Most of these loci are located on the *Francisella* pathogenicity island (FPI) [11], [12], [13].

Previously we identified a novel non- FPI encoded *F. tularensis* lipoprotein, encoded by locus FTT1103, that is defective in intracellular growth, and essential for virulence *in vivo* in the highly pathogenic *F. tularensis* subsp. *tularensis* strain Schu S4 [14]. We have designated the protein encoded by FTT1103 as FipB for *Francisella* infectivity potentiator protein B. FipB consists of a unique combination of conserved domains that are found in a class of virulence proteins called Mips (macrophage infectivity potentiator) [15], and DsbA oxidoreductases [16]. Mip is a homodimeric protein with peptidyl-prolyl cis/trans isomerase (PPIase) activity. Mip proteins are characterized by two conserved domains, the Forskolin-binding protein-N (FKBP-N), which is found at the amino-terminal end of Mip and also FipB, and FKBP-C, which encodes the PPIase activity. Mip protein was first identified in *Legionella pneumophila* as a virulence factor that was required for optimal intracellular survival and virulence *in vivo* [17]. Orthologs have been subsequently identified in several other Gram-negative bacteria in including *Coxiella burnetii*, *Neisseria gonorrhoeae* [18], and *Chlamydia* species [19]. A number of other Gram-negative bacteria have a Mip ortholog at least by bioinformatic annotation. *F. tularensis* has a Mip ortholog encoded by locus FTT1043.

Directly upstream of *fipB* in the Schu S4 genome is the FTT1102 locus, which we have designated as *fipA*. In the original annotation of the Schu S4 genome *fipA* was annotated as a pseudogene. However, proteomic analysis of LVS membrane fractions identified a peptide encoded by *fipA* [20]. The FipA protein is predicted to encode a 96 amino acid lipoprotein, and differs in only a single amino acid between Schu S4 and LVS. Like FipB, FipA shares some sequence and structural similarity to the FKBP-N domain. FipA and FipB are 28% identical to each other in a 54 amino acid overlap. FipA and FipB are both highly conserved (>98% identity) in all sequenced isolates and subspecies of *F. tularensis*.

Canonical DsbA proteins are periplasmic oxidoreductases that catalyze disulfide formation in nascent proteins in the periplasmic space [21]. The active site of DsbA is minimally defined by a Cysteine- any amino-acid-any amino acid- Cysteine (CXXC) motif embedded in a thioredoxin-like fold [22]. FipB contains a CXXC motif, and Straskova *et al.* have shown that recombinant protein of the LVS ortholog of FipB has oxidoreductase activity *in vitro* [20]. The Schu S4 and LVS FipB orthologs are highly similar; they differ in seven amino acid residues, which are scattered throughout the protein. LVS FipB also has 11 extra amino acids on its carboxyl terminus. There are at least eight families of DsbA-related proteins in the NCBI conserved domain database (www.ncbi.nlm.nih.gov/cdd). FipB is most similar to the DsbA_Com1_ like protein family (NCBI; conserved domain cd03023). Com1 is an outer membrane-associated protein of *Coxiella burnetii* [16]. Com1-like proteins are present in a number of gram-negative pathogens, but their roles as virulence factors or as oxidoreductases have not been fully explored.

The goals of this study were to determine if *fipA* had roles in intracellular replication and *in vivo* virulence, and determine whether the active site of FipB was involved in this protein's essential role in virulence. FipB is a novel protein for several reasons; it contains the conserved amino-terminal domain of Mip proteins, and as we show in this paper, it consists of three isoforms, and it has an accessory protein, FipA, that may function in post-translational modification. Here we show that FipA is not essential for virulence, though this mutant does not appear to replicate intracellularly. We have also shown that *in vivo* virulence is dependent on the CXXC motif of FipB. However, only the first cysteine of the CXXC motif is essential for FipB activity.

## Results

### *FipB* is *co-transcribed* with *fipA*

**Figure 1:**
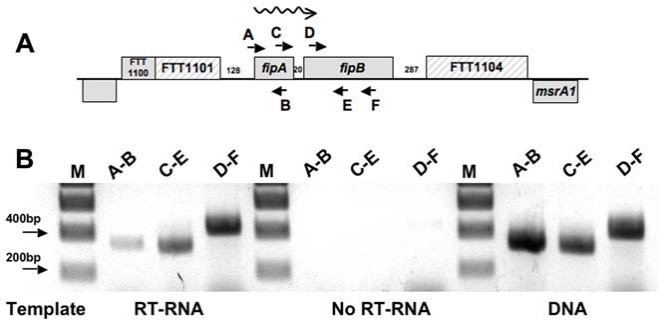
*fipA* and *fipB* are co-transcribed. RNA was isolated from wild-type Schu S4, and then converted to cDNA. (**A**) Overview of chromosomal region containing *fipA* and *fipB*. Short arrows indicate the location of the PCR primers. The wavy arrow indicates the direction of transcription. The numbers between the genes indicate the size of the intergenic region. The hatched boxes (FTT1101 and FTT1104) indicate predicted pseudogenes. (**B**) PCR was performed using the indicated primers (RT-RNA). Controls were: No reverse transcriptase added to the cDNA synthesis reactions (No RT-RNA), and PCR using Schu S4 DNA as template and the indicated primers (DNA). Expected sizes of PCR products were as follows A–B: 314 bps, C–E: 311 bps, and D–F: 392 bps.

**Figure 2:**
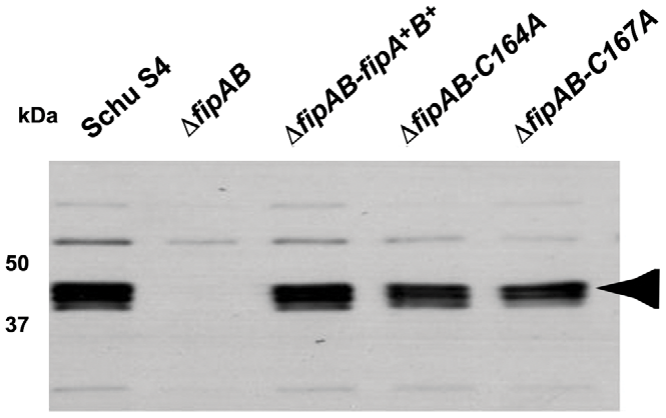
*In cis* complementation of Δ*fipAB*. Wild-type and mutated copies of *fipB* were introduced into Δ*fipAB* and then selected for integration into the *blaB* locus as described in methods. Western blots of overnight cultures were prepared and incubated with anti-FipB specific antibody. Arrow indicates the location of the FipB triplet isoforms.

Although there are only 20 base pairs separating the open reading frames of *fipA* and *fipB*, it was important for later complementation studies to confirm that these two genes were transcribed from the same promoter. We verified that *fipA* and *fipB* are co-transcribed by reverse transcription PCR (RT-PCR) (Figure 1). As shown in Figure 1, PCR products using primer pair C/E amplified the intergenic region between *fipA* and *fipB*, and primer pairs A/B, and D/F amplified intragenic fragments of *fipA* and *fipB*, respectively. It is possible that additional loci are co-transcribed with *fipAB*. *FipAB* is flanked by FTT1100, and predicted pseudogenes, loci FTT1101 and FTT1104. If FTT1101 consisted of an intact open reading frame, translation would terminate about 120 base pairs (bps) from the translational start of *fipA*. The predicted start of FTT1104 is 287 bps from the end of *fipB*. For *in-cis* complementation of *fipAB* we included 262 bps upstream of *fipA* in the plasmid used for constructing the complemented strains (Table 1, pAQ162, pAQ163, pAQ164). Since this region of DNA was able to drive expression of *fipB* in the complemented strains (Figure 2), it suggests that this region contains a promoter element.

**Table 1:** Bacterial strains and plasmids used in this study.

Name	Relevant characteristics	Source/Ref
*Francisella* strains		
Schu S4	*F. tularensis tularensis*, wild-type	CDC
BJM1031	Schu S4 Δ*fipB*	Qin [14]
BJM1068	Schu S4 Δ*fipA*	This study
BJM1069	Schu S4 *DfipA* ^+^ *B*	This study
BJM1076	*fipA^+^B^+^* in cis complement of Δ*fipAB*	This study
BJM1077	*fipA^+^B* C164A in cis complement of Δ*fipAB*	This study
BJM1078	*fipA^+^B* C167A in cis complement of Δ*fipAB*	This study
Plasmids		
pMP815	Chromosomal integration system vector	LoVullo [46]
pGIR463	*sacB* suicide vector	Sullivan [44]
pAQ136	5′flanking region of *fipA* in GIR463 (BM248/BM249)	This study
pAQ137	5′-and 3′flanking regions of *fipA* in GIR463 (BM250/BM251)	This study
pAQ138	5′-and 3′ flanking regions of *fipAB* in GIR463 (BM256/BM085)	This study
pAQ162	*fipA^+^fipB^+^* in pMP815	This study
pAQ163	*fipA^+^fipB* C164A in pMP815	This study
pAQ164	*fipA^+^fipB* C167A in pMP815	This study

### FipA affects post-translational processing of FipB

**Figure 3:**
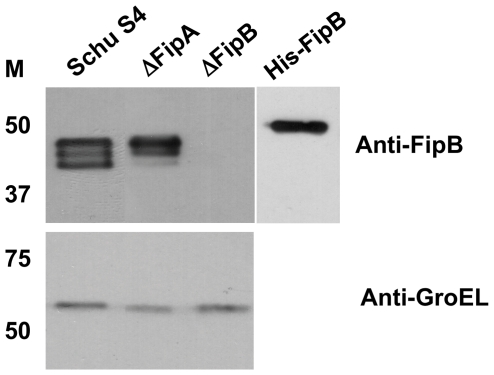
Detection of FipB in the Δ*fipA* bacteria. On Western blots FipB migrates as three bands. In the Δ*fipA* mutant the lower band was diminished. Western blot of bacterial lysates of indicated strains with anti-FipB antibody; antibody to *E. coli* GroEL, which cross-reacts with the *Francisella* protein, was used as a loading control. Recombinant His-FipB was used to generate the anti-FipB antibody, and serves as a positive control.

When Straskova *et al.* used various proteomic techniques to compare the protein profiles of wild-type LVS and an isogenic *fipB* (FTL_1096) mutant bacteria only two proteins were absent, FipB and FipA [20]. This suggested that FipB was required for FipA protein stability. To investigate the effects of FipA on FipB, and also the specific contributions of FipA to virulence, nonpolar mutants in Δ*fipA*, Δ*fipB*, and Δ*fipAB* were constructed. Western blots with anti-FipB antibody confirmed that the Δ*fipA* mutation was nonpolar (Figure 3). Anti-FipB specific antibody recognized three bands on Western blots. FipB has been identified as a glycosylated protein by carbohydrate detection and mass spectrometry techniques, so these three isoforms may reflect differences in glycosylation [23], [24]. All three bands disappeared in the Δ*fipB* mutant and also in the Δ*fipAB* mutant (data not shown). We noted that compared to the wild-type strain, the lowest migrating band of FipB was diminished in the *fipA* deletion mutant suggesting that FipA plays some role in FipB post-translation processing or modification such as glycosylation.

### Δ*fipA* mutant is defective in intracellular growth

**Figure 4:**
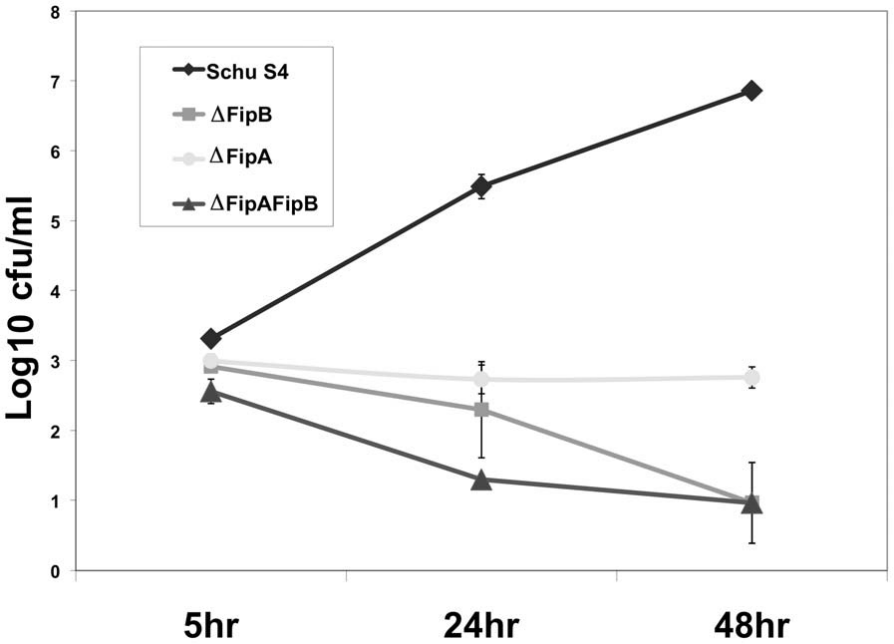
Δ*fipA* bacteria are defective in intracellular growth. J774A.1 cells were infected at an MOI of 50∶1 with the indicated strains of bacteria as described in materials and methods; cells were thoroughly washed, lysed at the indicated time points, and then diluted and plated to determine CFU/ml.

To explore and compare the roles of FipA and FipB in intracellular growth and virulence J774A.1 cells were infected with the *fipA*, *fipB*, or *fipAB* deletion strains, and then assayed for intracellular growth at several time points using gentamicin protection assays (Figure 4). All mutants exhibited statistically significant reduced growth when compared to wild-type Schu S4 at 5 hrs post-infection (p<0.001). The Δ*fipB*, and the double Δ*fipAB* mutants had similar phenotypes; in J774A.1 cells the number of CFUs recovered from these mutants at 24 hrs had decreased by about two logs from the 5 hr time point, while during this same time period Schu S4 CFUs had increased by almost 4 logs (Figure 4). In contrast, the Δ*fipA* mutant did not appear to replicate appreciably over this time period, and the number of recovered CFUs remained stable for up to 48 h. Similar patterns of defective growth for all of these mutant strains were also observed in A549 cells (data not shown). The difference in intracellular growth was not due to an inherent growth defect because all strains grew similarly in TSB/c and CDM media (data not shown). FipA and FipB may have independent functions in intracellular replication, but based on their sequence similarity, and altered levels of FipB isoforms in the *fipA* mutant, it seems likely that their roles in intracellular replication are linked.

### Δ*fipA* mutant is slightly attenuated *in vivo*

We have shown here and previously that *fipB* is essential for *in vivo* virulence in mice (Table 2) [14]. To test whether the intracellular growth defect of the *fipA* mutant would have any affect on virulence *in vivo*, C57BL/6 mice were challenged intranasally with 2800, 280, or 28 CFUs of Δ*fipA* bacteria. Despite a significant intracellular growth defect *in vitro*, the *fipA* mutant was only mildly attenuated *in vivo*; as few as 28 CFUs of the *fipA* mutant by an intranasal route was lethal, although there was 2–3 day delay in the time to death when compared to mice that received a challenge dose of 10 CFUs (Table 2).

**Table 2:** Survival of *fipA*, and Δ*fipAB* mice after intranasal inoculation.

Strain	Relevant genotype	# of mice	Inoculation dose (CFU)^1^	Days to Death^2^
Schu S4		4	10	5,5,5,5
BJM1068	Δ*fipA*	3	28	7,7,8
BJM1068	Δ*fipA*	3	280	6,7,8
BJM1068	Δ*fipA*	2	2800	5,6
BJM1069	Δ*fipAB*	4	7×10^7^	Survived >30 days
BJM1076	Δ*fipAB-fipA^+^B^+^*	3	10	6,6,7
BJM1076	Δ*fipAB-fipA^+^B^+^*	3	100	6,6,6
BJM1076	Δ*fipAB-fipA^+^B^+^*	3	1000	5,5,5
BJM1076	Δ*fipAB-fipA^+^B^+^*	4	4.3×10^4^	4,4,4,4
BJM1077	Δ*fipAB-fipA^+^fipB* C164A	4	8.8×10^7^	Survived >30 days
BJM1078	Δ*fipAB-fipA^+^fipB* C167A	4	3.4×10^7^	4,4,4,4
BJM1078	Δ*fipAB-fipA^+^fipB* C167A	4	3.4×10^4^	8,8,8,8
BJM1078	Δ*fipAB-fipA^+^fipB* C167A	4	270	9,9,9,10
BJM1078	Δ*fipAB-fipA^+^fipB* C167A	4	27	9,10,10, >20 days

1 C57/BL6 mice were intranasally challenged with the indicated inoculum dose, which was confirmed by plating the inoculum.

2 Indicates the number of days after challenge that mice showed the first signs of irreversible mortality, and were euthanized. Mice were followed for a minimum of 20 days. Similar to the Δ*fipAB* mutant, mice similarly challenged with the Δ*fipB* mutant survive for more than 30 days without any signs of infection [14].

### The conserved CXXC motif of FipB is required for intracellular replication

The CXXC motif has been shown to be critical for the enzymatic activity of *Escherichia coli* DsbA (EcDsbA), and also for substrate interactions [27], [28]. Therefore, we predicted that the CXXC motif of FipB would also be important for FipB's role in intracellular replication, and virulence. To investigate the importance of the CXXC motif in intracellular growth copies of *fipB* in which the cysteines in the CXXC motif had been replaced with alanines, (C164A and C167A), were integrated in to the *blaB* gene in a Δ*fipAB* strain along with the native promoter and wild-type *fipA*. Expression of the mutated genes was confirmed by Western Blots (Figure 2). The level of FipB in the *in-cis* complemented strains was similar to wild-type.

**Figure 5:**
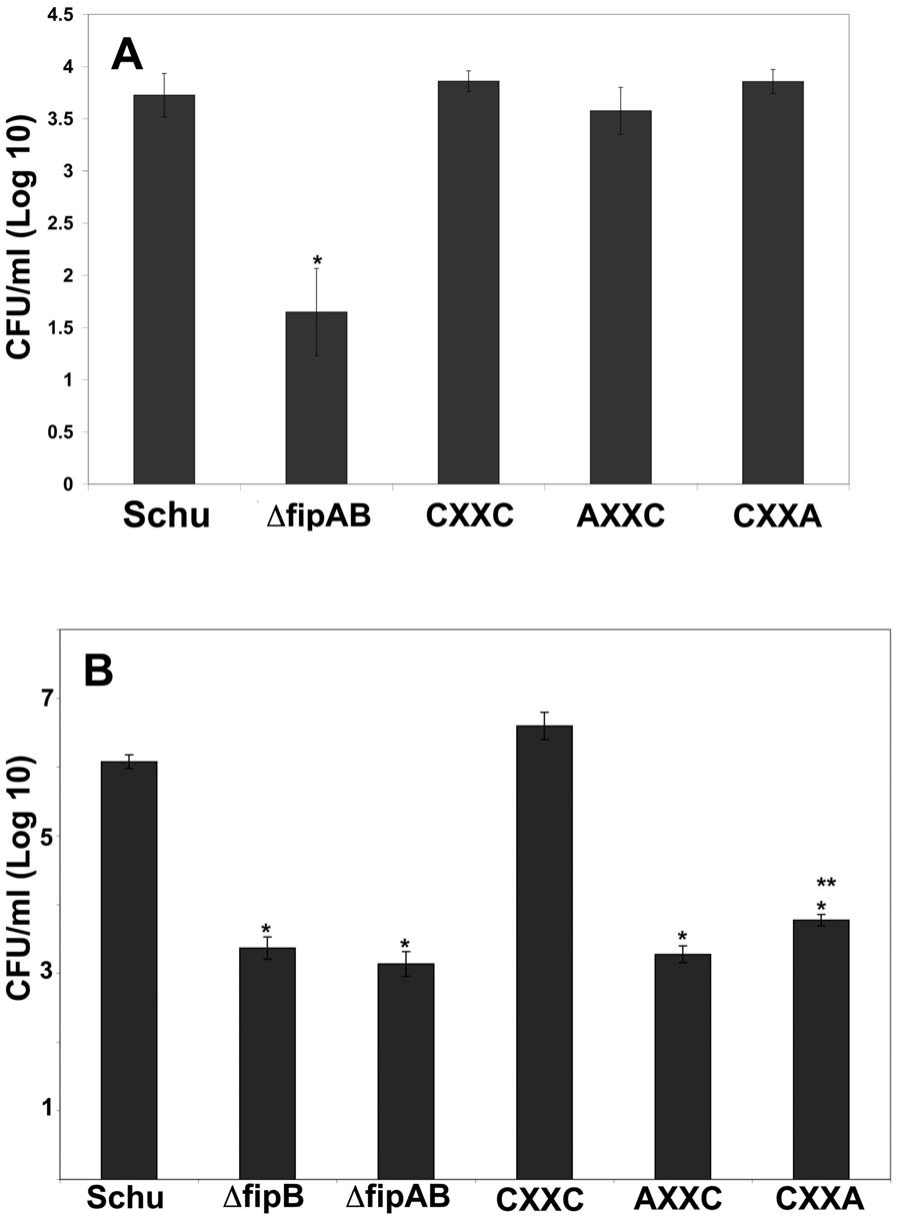
Effect of CXXC mutations on uptake and intracellular replication in J774A1. Monolayers of J774A.1 cells were infected with indicated strains at an MOI of 100∶1. The number of intracellular bacteria per well after infection for 2 (**A**) or 24 (**B**) hrs was determined as described in methods; cells were thoroughly washed, lysed at the indicated time points, and then diluted and plated to determine CFU/ml. Bars represent the mean ±SD of a representative experiment performed in triplicate. This experiment was repeated two times with similar results. For panel A “*” indicates p<0. 003 compared to Schu S4. For panel B “*” indicates p<0.00001 compared to Schu S4 and “**”<0.007 compared to **Δ** *fipAB*.

To examine uptake and the intracellular growth phenotype of the CXXC mutants, J774A.1 cells were incubated with the Δ*fipAB* or complemented strains, treated with gentamicin, and then assayed for growth at 2 and 24 hrs (Figure 5). At 2 hrs post-infection the Δ*fipAB* mutant had reduced uptake compared to the wild-type bacteria (Figure 5A). A Δ*fipB* mutant was also defective in uptake, but a Δ*fipA* mutant had uptake levels similar to wild-type bacteria (data not shown). Complementation with the C167A or C164A *fipB* genes (CXXA or AXXC, respectively) also restored uptake to wild-type levels. At 24 hrs post-infection complementation of the *fipAB* mutant with the wild-type *fipB* gene restored intracellular replication to wild-type levels. The numbers of bacteria recovered from *fipAB* mutant, or the strains complemented with CXXA or AXXC genes were significantly reduced compared to wild-type Schu S4 (p value<0.00001). However, when the complemented strains were compared to the Δ*fipAB* mutant, the number of CFUs recovered from the CXXA strain was statistically higher (p value<0.007) than the Δ*fipAB* mutant, while there was no statistical difference between the AXXC and Δ*fipAB* mutants. These results indicated that the CXXC motif was not required for uptake, but was important for FipB mediated intracellular replication, however, the C164 amino acid was more critical for function.

### The conserved CXXC motif of FipB is required for *in vivo* virulence

In the *fipB* and *fipAB* deletion mutants a defect in intracellular growth correlated with avirulence in mice ([14] & Table 2). However, this paradigm was not true of the Δ*fipA* mutant; despite a significant intracellular growth defect this mutant still retained virulence. One difference between Δ*fipA*, and Δ*fipAB*, was that between 24 hrs and 48 hrs post-infection the number of viable Δ*fipA* bacteria was stable, while viable Δ*fipAB* bacteria had decreased in number. To test whether the increased number of bacteria recovered from the CXXA complemented strain, compared to the Δ*fipAB* strain, translated to a difference in virulence *in vivo*, mice were challenged intranasally with decreasing doses of these various strains (Table 2). Similar to the parental deletion strain, the AXXC mutant appeared to be avirulent; mice survived challenges of 8.8×10^7^ CFUs for more than 30 days. The CXXA mutant was also attenuated but still retained virulence; mice challenged with 3.4×10^4^ CFUs died on day 8. Mice challenged with 10 CFUs of Schu S4 died on day 5 post-infection, but few as 27 CFUs of the CXXA, with one exception, also resulted in a fatal infection on day 9 or 10 post-infection. These results were consistent with the intracellular replication data (see Figure 4), and support a critical requirement for the first cysteine in the CXXC motif of FipB for virulence.

## Discussion

FipB is a novel lipoprotein that is required for uptake, intracellular replication and *in vivo* virulence of *F. tularensis*. Although FipB is novel it has two conserved domains, DsbA_Com-1_like (cd03023), and FKBP-N, the amino-terminal domain of FKBP-type peptidyl prolyl isomerases (cl03173), which are also known as Mip proteins. To date, this combination of conserved domains is unique to FipB. FipB is also a lipoprotein, which is unusual, but not unique for DsbA proteins. Most DsbA are thought be periplasmic proteins, but a few lipoproteins have been identified; *Neisseria sp.*, have three DsbA orthologs, and two of these are lipoproteins [29]. The other novel aspects of FipB include the presence of three isoforms, which are likely due to post-translational modification [24], and the presence of an accessory protein, FipA.

In the Schu S4 genome the FTT1102/*fipA* locus was annotated as a pseudogene. However, consistent with the proteomic data from Straskova *et al.* [20], which detected the 96 amino acid peptide corresponding to FipA, we have shown that *fipA* is indeed a functional locus. Deletion of *fipA* resulted in a significant intracellular growth defect *in vitro*, though the impact of this gene loss was not appreciably significant *in vivo*. It is possible that Δ*fipA* bacteria can replicate in other cell lineages that we have not tested. Horzempa *et al.* found that a Schu S4 *pyrF* mutant, which could not replicate in human primary macrophages, was able to replicate in HEK-293 and was virulent in mice [30]. Our results also suggest that replication in macrophages is not an essential requirement for virulence.

FipA also has similarity to the FKBP-N domain and is predicted to be a lipoprotein. One could speculate that *fipA* and *fipB* arose as gene duplications, and then *fipA* was truncated either in the process or subsequently. However, this event would have to have been an early event in the speciation of *Francisella*, because FipA is highly conserved among the various species and subspecies of *Francisella*, including the more distantly related *Francisella philomiragia*. There has also been considerable sequence divergence between FipA and FipB, which are only ∼28% identical at the amino acid level. The conserved domain FKBP-N, which is shared by the two proteins, has previously only been found in the amino-terminal region of Mip proteins. The crystal structure of LpMip has been determined [15]. Mip forms a dimer [31], and has its two conserved domains connected by a very long alpha-helix. The C-terminal domain of Mip (100–213 amino acids) has PPIase activity, and amino terminal FKBP-N domain contains the alpha helical N-term portion of the protein, which is required for dimerization [15]. The LpMip protein has a complex role in virulence. It is required for optimal replication in human macrophages and amoebae [17], migration through an epithelial barrier [32], and secretion of a phospholipase C-like activity in culture supernatants [33]. It has been reported that the FKBP-N or dimerization domain, but not the PPIase domain, is required for full virulence in *Acanthamoeba castellanii* [34], suggesting that dimerization and PPIase domains have separable functions. One model for FipA function is that it physically interacts or dimerizes with FipB, which then stabilizes a conformation state that facilitates post-translational modification or processing. This model is consistent with our observation that the level of one of the isoforms of FipB was diminished in a FipB mutant. FipA dimerization with FipB could also enable a different functional role for FipB. By proteomic analysis of membrane fractions Straskova *et al.* could not detect FipA in a FipB mutant [20], so FipA may be unstable unless it is able to associate with FipB. The presence of the FKBP-N dimerization domain in FipB also suggests that FipB could form a homodimer, which FipA could potentially regulate or influence. This will require further investigation. We have observed higher molecular sized complexes on nonreducing gels, but these complexes were sensitive to reducing agents, and are likely artifactual interactions between the cysteines in the active site.

The other conserved region of FipB, the DsbA-Com1-like domain, is one of several DsbA-related conserved domains. DsbA was first identified in *E. coli*, and is the best characterized DsbA protein both biochemically, and structurally [28]. The conserved CXXC motif, present in most all DsbA-related conserved domains, is critical for the oxidoreductase enzymatic activity of DsbA. In our studies we found that the CXXC motif was critical for FipB's role in intracellular survival and *in vivo* virulence, but does not appear to be essential for bacterial uptake. In the intracellular growth assays mutation of two cysteines individually did not produce identical results; the first cysteine residue was essential, while the second cysteine mutant had only reduced function. This finding is consistent with functional characterization of the CXXC motif of EcDsbA. Based on biochemical and crystallographic studies, the first cysteine is the nucleophilic residue that forms a mixed disulfide bond with its substrates. The second cysteine is hidden within the molecule and less accessible to solvent. One study that illustrates this difference examined the oxidation of beta-lactamase, a substrate of EcDsbA. When a CXXS mutant of EcDsbA was expressed in a wild-type strain it acted as a dominant negative mutant, which produced a decrease in the oxidation of beta-lactamase [35]. However, addition of oxidized glutathione to the media restored beta-lactamase folding. A SXXC mutant expressed in a wild-type strain did not exhibit a similar dominant negative phenotype. Based on this model, our *in vivo* experiments suggest that the *in vivo* environment must be sufficiently oxidizing so that the CXXA FipB mutant is able to carry its function at a level that is sufficient to promote virulence.

It is likely that at least part of FipB's role in virulence is through the folding of substrates that have critical roles in virulence. In other pathogenic bacteria DsbA is important for the structure or function of a number of virulence factors including the biogenesis of type IV pili in bacteria such as EPEC *E. coli*, and *Pseudomonas aeruginosa* [36], [37], the assembly and function of type III secretion systems in *Salmonella typhimurium* and *Shigella flexneri* [38], [39], [40], and the Dot/Icm type IV secretion system of *L. pneumophila* [41]. Identifying FipB substrates will help to define the essential elements of *F. tularensis* pathogenicity. However, proteins that contain a conserved DsbA pfam motif can be quite diverse [42]. With the exception of the active site and a few other conserved amino acids, many share very little additional sequence similarity. Therefore, it is likely that the function or structure of some proteins that contain the DsbA pfam is different, narrowed, or expanded. A number of bacteria have more than one DsbA-related protein, which also suggests more specialized functions [42]. *Salmonella* typhimurium, for example, contains plasmid encoded protein SrgA, which is a DsbA-related protein that has a restricted substrate specificity for the plasmid encoded fimbriae PefA [43]. The usual features of FipB, which include the FKBP-N domain and some interaction with FipA, suggest that FipB may also have specialized roles in virulence. The observation that complementation with CXXA and AXXC alleles was able to restore uptake supports a specialized role for FipB. FipB may act as a chaperone, or perhaps more directly mediate this activity. Indentifying these roles will help to define the essential aspects of *F. tularensis subsp. tularensis* virulence.

## Materials and Methods

### Ethics Statement

All experimental procedures and care of animals was approved by the University of Virginia's Institutional Animal Care and Use Committee. The University's Animal Welfare Assurance number is Animal Welfare Assurance #A3245-01, and the vivarium is accredited by the Association for Assessment Accreditation of Laboratory Animal Care International.

### Bacterial strains, primers, plasmids and culture

Bacterial strains, plasmids, and primers used in these experiments are listed in Tables 1 and 3. Plasmids pGIR463 and pMP815 were kind gifts of Girija Ramakrishnan and Martin Pavelka, respectively. *E. coli* strains were grown in Luria-Bertani (LB) broth or on LB plates with kanamycin (50 µg/ml) or ampicillin (100 µg/ml) when required. *F. tularensis subsp. tularensis* (type A) Schu S4 was cultured on cysteine supplemented Muller-Hinton agar (MHA/c) or in cysteine supplemented Trypticase Soy broth (TSB/c) [44]. For *F. tularensis* strains 15 µg/ml of kanamycin was added when appropriate. Studies involving Schu S4 and derived strains were carried out in an approved Biosafety Level 3 laboratory.

**Table 3:** Primers used in this study.

Primer	Sequence 5′-3′	Descript.	Restr. Enz.
BM063	TCCATATGCAAGAAATGGCTGCTC	F *fipB*	*NdeI*
BM064	GCGGCCGCTATAAGAAGGATAGGC	F *fipA*	*NotI*
BM066	GGATCCTATCATCATCTTGGCTGAGC	R *fipB*	*BamHI*
BM150	CTTTGATTATCAAGCTATGTACTGTTCTAAGCTTGC	F *fipB* C164A	
BM151	GCAAGCTTAGAACAGTACATAGCTTGATAATCAAAG	R *fipB* C164A	
BM152	CAATGTATGTACGCTTCTAAGCTTGCTTGCTCC	F *fipB* C167A	
BM153	GGAGCAAGCAAGCTTAGAAGCTTACATACATTG	R *fipB* C167A	
BM245	AGAAAATATGCGGCCGCGAAATAATAGGAG	F *fipA*	*NotI*
BM208	TCCTCGAGCTTATTTCTTTTGAGCAGCC	R *fipA*	*XhoI*
BM248	GAGCCCTAGGTAGAACAATGGCAACAGG	F *fipA* 5′deletion	*AvrII*
BM249	AGGCGGCCGCATTATTTAGTTTCTCCTA	R *fipA* 5′deletion	*NotI*
BM250	ATAGATCAGCGGCCGCATGCAATGATTGAATTCC	F *fipA* 3′deletion	*NotI*
BM251	ATTAGAGCTCAACACTATCATCATCTTGGCTGAGC	R *fipA* 3′deletion	*XhoI*
BM256	CTAAGTCTGCGGCCGCAACAACTAGTACTAGC	F *fipAB* 5′deletion	*NotI*
BM085	CTCGAGATTACAGCATTACCAGCTGC	F *fipAB* 3′deletion	*XhoI*
BM297	GAGGAATAATAAATGAAATTAACTAAAACTCT	F *fipA*	
BM298	TTATTTCTTTTGAGCAGCCAT	R *fipA*	

### DNA manipulation, cloning, and transformation

DNA was prepared and purified using a commercial kit (Qiagen, Valencia, CA). Oligonucleotides were synthesized by Integrated DNA Technologies Inc. (Coralville, IA). Restriction endonucleases and ligase were purchased from New England Biolabs (Ipswich, MA). HotStart® Taq (Qiagen) was used for routine PCR. FastStart® High fidelity PCR system (Roche, Indianapolis, IN) was used for construction of complementary and suicide plasmids. All cloning products were verified by DNA sequencing, which was performed at the University of Virginia Biomolecular Research Facility. Site direct mutagenesis was accomplished with a site-directed mutagenesis kit (QuikChange®, Agilent Technologies, Cedar Creek, TX) using primer pairs BM150/BM151 or BM152/153, and pAQ038 as template. Expression of *fipB* and mutant genes was verified by Western blot with rabbit anti-FipB antibody (1∶10,000) [14]. DNA transformation was performed as previously described [44].

### Construction of deletion and integration plasmids

To confirm the non-polarity of our deletion mutants we first complemented these mutations using the *Francisella* shuttle vector, pFNLTP [25]. However, *in-trans* complementation only partially restored the intracellular growth defect, and these strains also grew poorly in liquid culture (data not shown). We hypothesized that over-expression of *fipB* was deleterious. To circumvent this problem we integrated the *fipAB* genes, along with the 262 bps upstream, into the *blaB* locus using the plasmid developed by Lovullo *et al.* [26]. To construct in-frame deletions of *fipA* and *fipAB* PCR products corresponding to regions upstream and downstream of *fipA* or *fipAB* were produced with primer pairs BM248/BM249 and BM250/BM251 for *fipA* and BM256/BM085 for *fipAB* (Table 3), and then cloned into the *sacB* suicide vector pGIR463 [45]. Plasmids for *in cis* complementation of *fipA*, and *fipAB*, and the CXXC mutants were produced by PCR amplification of the genes using the primers listed in Table 2. Each amplicon contained 262 bp of the sequence upstream of *fipA*. PCR products were ligated into the *blaB* region of pMP815 [26]. The resulting plasmids were introduced into the appropriate host strain, and integrants or subsequent gene deletion mutants were selected as previously described [14]. The nonpolarity of the *fipA* deletion was verified by the detection of FipB on Western blot with anti-FipB antibody.

### Reverse Transcription PCR

Total RNA was isolated from overnight cultures using an RNeasy Protect mini kit (Qiagen) and treated with DNase I (Qiagen) to remove contaminating genomic DNA according to the manufacture's instructions. First strand cDNA was generated using SuperScript II Reverse transcriptase (Invitrogen) and random primers. A parallel transcription reaction without the reverse transcriptase enzyme was conducted to control for DNA contamination. Two µl of each reaction was used as a template for 50 µl PCR reaction.

### Uptake and Intracellular growth assays

Assays were performed with murine macrophage J774A.1 (ATCC#TIB-67) cells propagated in high glucose DMEM supplemented with 10% fetal bovine serum. Cells (2.5×10^5^/well) were seeded in 24 well plates, and incubated at 37°C, 5% CO2 for 18 h. Fresh cultures of *F. tularensis* were diluted in cell culture medium to reach the desired multiplicity of infection (MOI). Actual inoculum amounts of bacteria were determined by plating serial dilutions of the culture inoculum. The plates were centrifuged at 800×*g* for 8 min to start the infection, and then incubated at 37°C for l h. Cells were washed three times in PBS, and then extracellular bacteria were killed by gentamicin treatment (50 µg/ml). At the assay endpoint cells were washed, and then lysed with 0.1% sodium deoxycholate. Lysates were diluted and plated to determine the number of colony form units (CFU) in each well. Each experiment had triplicate wells and repeated a minimum of two times.

### Production of FipB antiserum

The DNA sequence of *fipB* gene was resynthesized with a his-tag and codons that were biased for *E. coli* expression (Accession #JN120022), and then cloned in pET Universal [46]. The expression of recombinant *fipB* was induced by the addition of 1 mM IPTG to log phase bacteria. The protein was purified from induced lysates using Talon beads (Clontech) and eluted with imidazole according to manufacturer's recommendations. Purified protein was dialyzed against PBS, and protein concentration was determined by BCA assay (Pierce). Purity was verified by SDS-PAGE and Western blots. Rabbit anti-his-tagged FipB serum was prepared by Covance Research products Inc (Emeryville, CA). Mouse anti-FipB serum was made in house.

### Mouse virulence studies

For intranasal inoculation 8 to 10-week-old C57BL/6 mice (Jackson Laboratory) were anesthetized with ketamine-HCl-xylazine. Twenty microliters of bacteria or PBS was inoculated into the nares. The actual inoculation doses were confirmed by viable plate counting. The mice were monitored daily. Mice were humanely euthanized when death was considered to occur within 24 h. The University of Virginia's Animal Care and Use Committee approved all mouse studies.

### Statistical analysis

All values were expressed as Mean ±SD and evaluated by using Student's unpaired Two-tailed *t* test with log transformed data, and assuming unequal variance.
